# Ultra-sensitive graphene sensor for measuring high vacuum pressure

**DOI:** 10.1038/s41598-017-13038-3

**Published:** 2017-10-03

**Authors:** Sung Il Ahn, Ju Ra Jung, So Young Choi, Min Hwa Son, Yu Jin Hong, Jung-Chul Park

**Affiliations:** Division of Energy and Chemical Engineering, Silla University, Busan, 617–736 Republic of Korea

## Abstract

We demonstrate here that several different graphene nanoribbon (GNR) samples can be separated from the GNR mixture synthesized by conventional methods. The sheet resistance of the purified GNR gradually decreased with decreasing pressure at 30 °C, whereas it increased at 100 °C. A hypothesis based on van der Waals attractive interactions between GNR sheets was introduced to explain this finding. This hypothesis verified by the shifted main peaks in vacuum X-ray diffraction spectra: 0.022 nm and 0.041 nm shifts were observed for reduced graphene oxide (RGO) and GNR, respectively. Theoretical calculations indicated that, for RGO, the shifted distance was similar to the calculated distance. The response of the GNR sensor to pressure changes occurred rapidly (in seconds). The normalized response time of each sample indicated that sensor using GNR reduced the tailing of the response time by shortening the diffusion path of gas molecules. The sensitivity of the GNR sensor was three times that of RGO in the given pressure range. Moreover, the sensitivity of GNR was much larger than those of the most popularly studied pressure sensors using Piezoresistivity, and the sensor could detect vacuum pressures of 8 × 10^–7^ Torr.

## Introduction

Many pressure sensors using graphene and its derivatives have been studied such as field emission pressure sensors,^1^ a graphene squeeze-film pressure sensor,^2^ and micro-electro-mechanical systems piezoresistive pressure sensors^3–8^ which determine the strain produced in graphene by an external pressure. Most previous reports on pressure sensors have focused mainly on high pressure measurements. Except for a few cases, sensors have rarely been applied for the measurement of pressures below 1 Torr.

Vacuum technology is a generalized technology used in many industries and scientific research, such as those related to semiconductors, surface engineering, and space science. Thus, the precise measurement of the vacuum pressure is critical and fundamental for advanced research and production. Several vacuum gauges that are generally adopted in industries are shown in Figure S1. The capacitance gauge, a direct vacuum gauge, allows the most precise measurement of vacuum pressure by the measurement of differential pressure between the vacuum and ambient pressure. However, the measurable vacuum range of this gauge is limited to only three or four orders of magnitude of the vacuum range (for example, 10^−3^ to 1, 1 to 1000, or 10^–1^ to 100 Torr), and pressures below 10^−5^ Torr can hardly be measured. The next most popular gauge is the thermal-conductivity-type indirect vacuum gauges and ionization-type gauges, which are used in the pressure ranges from 1 to 10^−4^ Torr and from 10^−2^ to 10^−12^ Torr, respectively. The measuring errors of these gauges are remarkably high, approximately 30% for the thermal-conductivity-type gauge and more than 10% for the ionization gauges. In addition, the size and power consumption of present sensors in the gauges are too large for these devices to be used for wireless measurements, or measurements for isolated thin and small devices. For example, current vacuum gauges cannot directly measure the speed of diffusion of gas molecules from outside to inside a device through a protective layer, or devices isolated from the atmosphere including display devices.

We have previously investigated the use of Van der Waals (VDW) attractive force between reduced graphene oxide (RGO) sheets for application in a vacuum pressure sensor.^9^ Compared with the sizes of current vacuum sensors, the sensor using RGO can have reduced dimensions of only a few μm^3^ in volume based on the active area of the sensor. Moreover, an RGO-based vacuum pressure sensor using VDW force would offer high durability and low power consumption because it measures the resistance change of the RGO based on its nano-scale physical motion against vacuum pressure.

**Figure 1:**
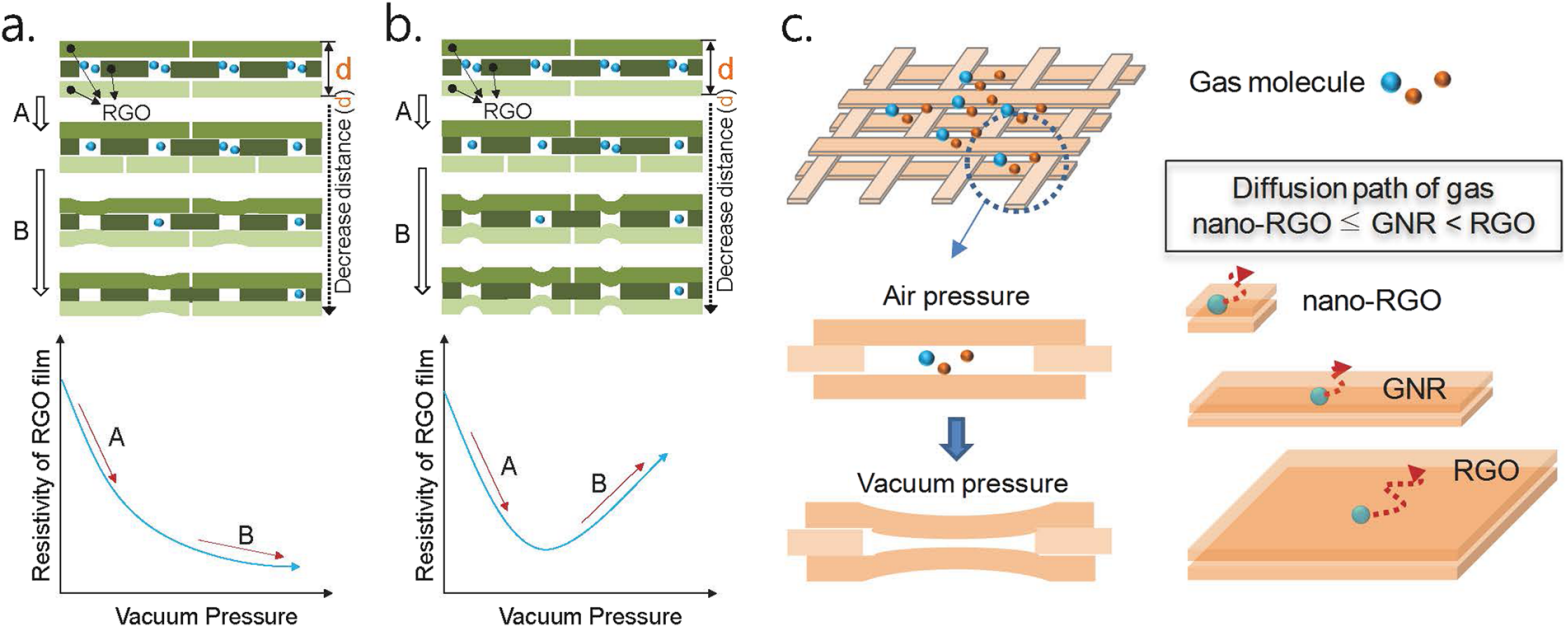
Expected behaviors of RGO sensors against vacuum pressure. (**a**) A first order change of RGO resistance occurred at low temperature mainly due to the wide VDW interactions between RGO sheets, (**b**) A second order change of RGO resistance occurred at high temperature mainly due to the local VDW interaction, and (**c**) Description of sensor action of GNR (note that the actual GNR film can have various pore structures, as shown in the TEM images of concentrated GNR samples in Figure S2).

VDW forces, especially, attractive interaction between molecules, are responsible for many of the physical properties of carbonaceous compounds such as carbon nanotubes (CNTs),^10,11^ graphite, and graphene and its derivatives. The VDW attractive force is generally too weak to attract small molecules because of the large kinetic energy of the molecule, whereas very large carbonaceous compounds are greatly affected by the VDW force, even at high temperature. Based on the VDW force and thermal vibration modes of a graphene sheet,^12,13^ we propose two different physical motions of carbon clusters on adjacent RGO sheets with voids in a film and thereby electrical responses against vacuum pressure (See Fig. 1a and b). Supposing that RGO sheets in a film are ideally coated and aligned, the most probable size of the void in the vertical direction will be approximately of the size of one sp^2^ carbon atom. In addition, smaller or larger voids sizes can be formed by defect structures of RGO sheet and random stacks of the RGO sheets. Because the VDW attraction force appears below the distance of 2.5 × σ (one of the Lennard−Jones parameters, 0.34 nm for graphite),^10^ this force affects carbon clusters in RGO sheets located above and below the voids in the film. However, the distance from these carbon clusters to other carbon clusters is maintained because of the gas molecules in the voids of the film. A decrease of the number of gas molecules to a certain content results in a decrease of the distance between RGO sheets because of the VDW interaction, thereby resulting in an increase of the conductivity of the RGO film. A further decrease of the gas contents may result in various motions of carbon clusters or the RGO sheet due to the VDW interaction, vibration energy, and various elastic forces depending on the shape and size of the RGO sheet. At low temperatures, the spacing of the RGO sheets decreases over a wide range because the vertical oscillation of the carbon clusters on the RGO sheet is too small to deform the cluster by VDW interaction (See Fig. 1a). In contrast, at high temperature, VDW attractive interactions can occur locally rather than over a widely area because of the large vibration distance of carbon clusters (See Fig. 1b).

According to a report on the vibrational modes of graphene, micro-sized graphene sheet can allow local fluctuations of carbon clusters, whereas a nano-sized sheet including graphene nano-ribbon (GNR) allows different vibrational motions of carbon clusters.^12,13^ Based on this finding, we can speculate that nano-sized graphene has a different sensor property depending on its dimension. As observed in Fig. 1c (note that the actual GNR film can have various pore structures, as shown in the TEM images of concentrated GNR samples in Figure S2.), nano-sized graphene also has an advantage in the diffusion of gas molecules compared with micro-sized graphene and allows faster response to changing pressure. In this study, we fabricated sensor devices using various types and sizes of graphene samples and investigated the electrical behavior against vacuum pressure. We also presented evidence for the VDW interaction in the samples using X-ray diffraction (XRD) spectra measured under vacuum at various temperatures. In a previous study, there was no direct evidence for the vacuum pressure-sensing principle of RGO (or intercalated RGO). Instead, theoretical calculations based on experimental results were provided to support the sensing principle of RGO. In addition, when synthesizing GNR from MWCNT, we demonstrated that the as-synthesized GNR mixture containing a small quantity of CNTs could be purified by simple pH control of the mixture.

## Results and Discussion

**Figure 2:**
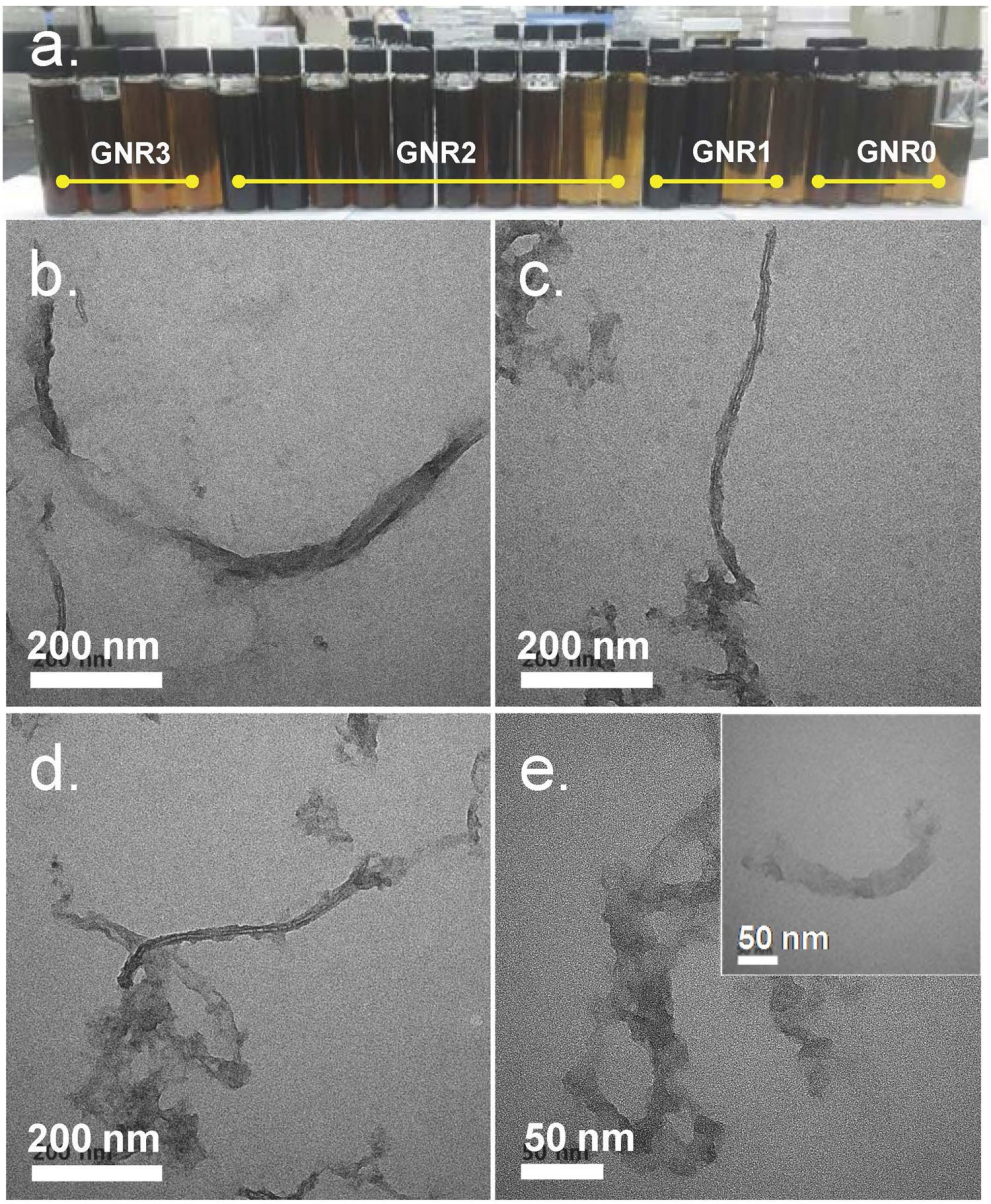
Separated GNRs and their TEM images. (**a)**. A photographic image of separated GNR samples. TEM images of (**b**). GNR0, (**c**) GNR1, (**d**) GNR2, and (**e**). GNR3 (Inset is an isolated GNR sheet). See also Supplementary Fig.S1; TEM images of concentrated GNR samples.

**Figure 3:**
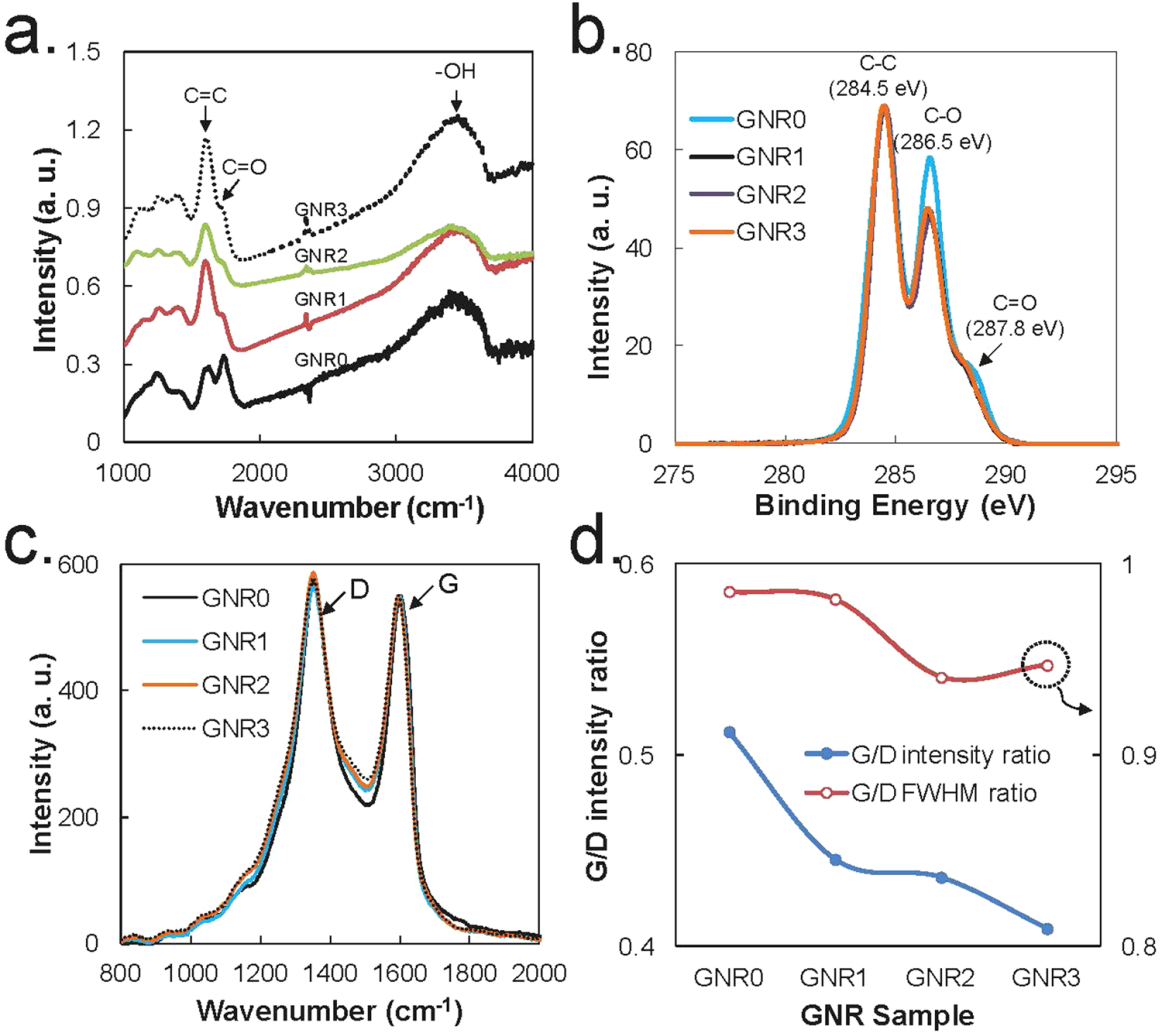
Characterizations of GNR samples. (**a**) FT-IR spectra, (**b**) XPS spectra, (**c**) Normalized Raman spectra of GNR samples, and (**d**). The G/D intensity and FWHM ratios of each GNR sample obtained from the curve fittings of (**c**).

The synthesis methods of GNR have been reported in many studies. Among these, we chose the method using KMnO_4_ and phosphoric acid as unzipping agents of CNT.^15^ During the synthesis of GNR using the method, we observed that several different GNR samples could be separated by controlling the pH of the final reaction mixture likely because of their size and oxygen content. As shown in Fig. 2a, various types of GNR sample were successfully isolated from the same mixture obtained by the oxidation reaction of MWCNTs. The TEM images of the GNR samples in Fig. 2b–e demonstrate that GNR0 comprised CNTs, several peeled carbon layers from MWCNTs and GNRs. GNR1 and GNR2 mostly comprised a ribbon-shaped graphene and a small amount of tubes. GNR3 also has a ribbon shape, but it is smaller in size than other GNR samples and has almost no tube shape (See also Supplementary Figure S2; TEM images of concentrated GNR samples). The FT-IR spectra of the GNR samples in Fig. 3a demonstrate that the samples had similar oxygen group contents except for GNR0. The normalized XPS spectra of the samples in Fig. 3b clearly indicate that GNR0 contains more C-OH and C = O groups than the other GNR samples. From GNR1-GNR3, we hardly detected any differences in their chemical structures in the XPS and FT-IR spectra. In contrast, the Raman spectra in Fig. 3c–d clearly show a reduction in the graphite band (G band) compared with the D band due to the defects or oxidized sites on the GNR sheet. Several reasons can be considered to explain the formation of different GNRs in terms of chemical structure and size. First, the oxidation of MWCNTs using KMnO_4_ can open the CNTs of MWCNTs in longitudinal and radial directions.^16,17^ Second, considering that GNR is detached one by one from the starting material of MWCNTs when the oxidative cleavage reaction is being performed, various lengths of GNRs can be obtained depending on the diameter of the CNT layer in the MWCNTs. Third, the early detached GNRs have more opportunities to react with an oxidant than the GNRs detached later. In addition, the detached GNR had edge defects (or oxidized sites) that could easily react with an oxidant compared with the un-reacted sites on GNR sheets or MWCNTs. An important and interesting result for the separation process of GNRs is that GNRs with similar oxygen group contents can be further separated under a certain pH. This finding indicates that fine control of the pH can yield more purified GNR using the process proposed in this experiment.

**Figure 4:**
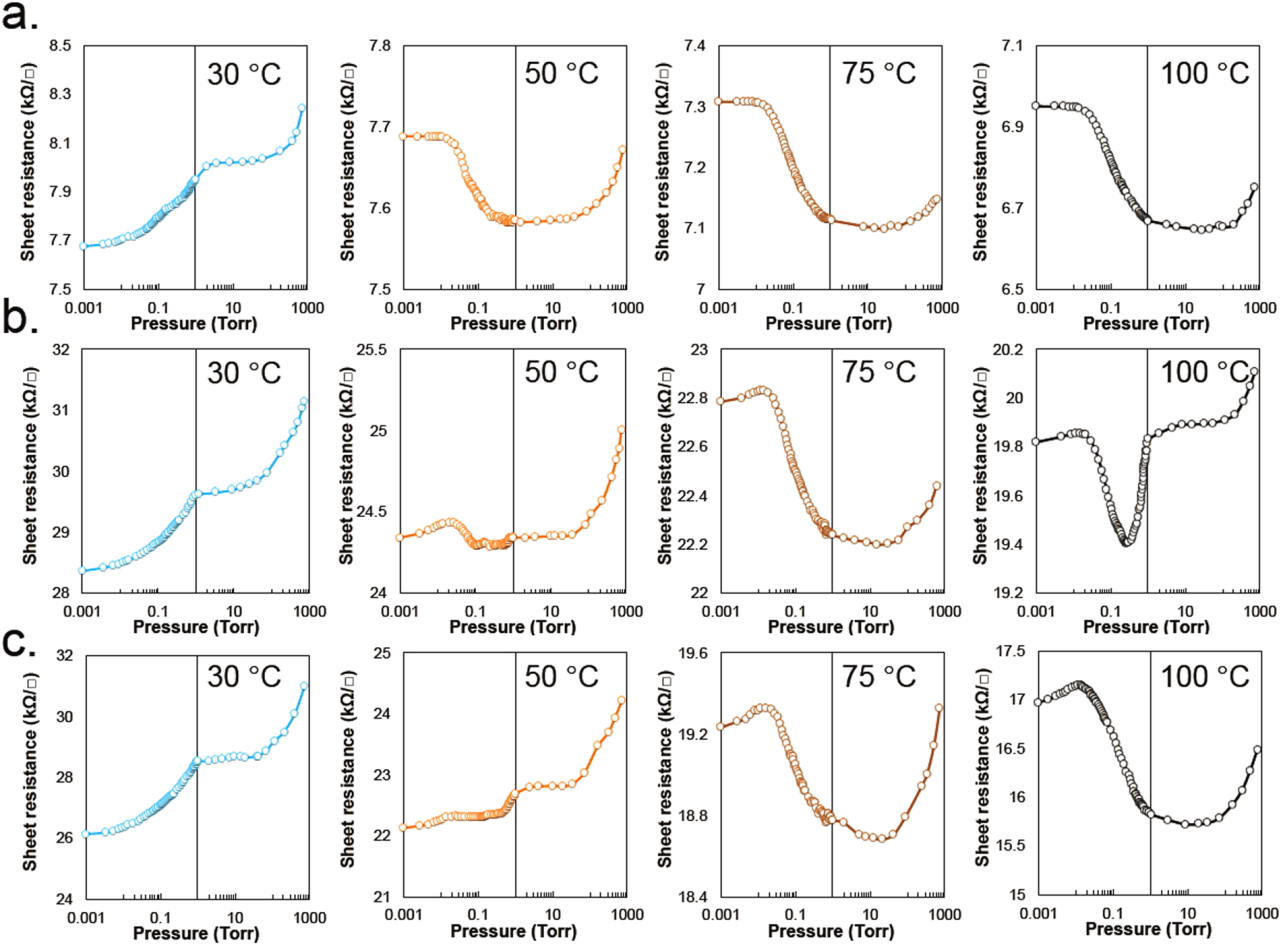
Sheet resistances of the samples against vacuum pressures ranging from 10^–3^ Torr to air pressure: (**a**) RGO, (**b**) GNR0, and (**c**) GNR3 at 30 °C, 50 °C, 75 °C, and 100 °C.

The sensor activities of the GNR samples were evaluated by measuring the sheet resistance against a vacuum pressure at 30, 50, 75, and 100 °C from 10^−3^ Torr to ambient pressure using a four-point probe placed in a vacuum chamber. The change of resistance with vacuum pressure showed completely different trends depending on temperature. At 30 °C, the sheet resistance for all the samples decreased with increasing vacuum pressure. The sheet resistance plots comprise complicated shapes at 50 °C and parabolic curves at 75 °C and 100 °C, as observed in Fig. 4.

**Figure 5:**
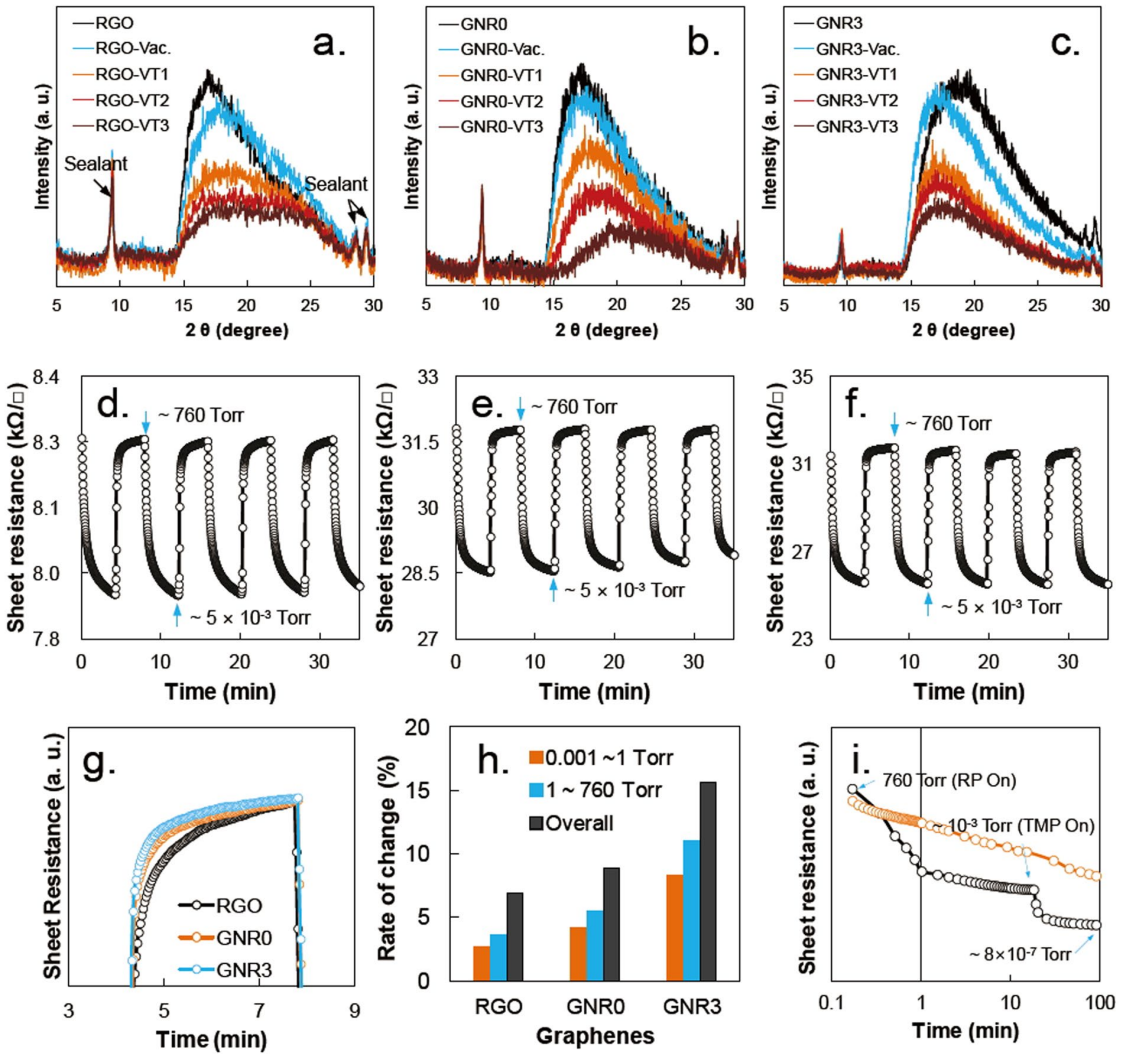
XRD spectra obtained under vacuum (approximately 0.3 Torr) at 18 °C (-Vac.), 40 °C (-VT1), 70 °C (-VT2), and 100 °C (-VT3): (**a**) RGO, (**b**) GNR0, and (**c**) GNR3. Response time of GNR sensors at 30 °C: d. RGO, (**e**) GNR0, and (**b**). GNR3. (**g**) Comparison of response time of each sample after the normalization of the sheet resistance. h. Rate of sheet resistance change of the samples measured at 30 °C. i. Normalized resistance changes of RGO and GNR3 samples between ambient pressure to approximately 8 × 10^–7^ Torr at 17 °C.

Similar to the RGO sheet, the electrical response of GNR film can be interpreted by VDW attractive interactions. VDW attraction generally begins at distances less than 2.5 × σ (0.85 nm for graphite, corresponding to 2θ ≈ 7.5°), as mentioned in the Introduction, and reaches a maximum near the VDW radius of the carbon atoms. For GNR film, the distance between sheets is much shorter than 0.85 nm based on the XRD spectra in Fig. 5a-c. Thus, we can expect that most of the GNR sheets in a film attract each other by VDW force. However, under air pressure, gas molecules in the film interrupt the approach of the GNR sheets to adjacent sheets. Decreasing the pressure to a certain point (between approximately 10 and 1 Torr) allows the GNR sheets to approach each other, leading to a decrease of the sheet resistance due to the VDW attractive interactions (See Fig. 1a). Moreover, further reducing the pressure results in a different electrical behavior of the GNR film that is dependent on temperature. As observed in Fig. 4, the sheet resistance of the GNR film gradually decreased with increasing vacuum pressure at 30 °C and below approximately around 1 Torr, whereas it increased at 100 °C. To understand the sheet resistance change as a function of pressure, we should consider the conflicting role of the vibrational energy of the samples. Vibrations in the GNR sheet can reduce the attractive forces between adjacent carbon clusters, but can also reduce the distance between adjacent GNR sheets locally or widely. The decreasing distance between carbon clusters on adjacent GNR sheets causes an exponential increase of the VDW attractive interaction. Therefore, the electrical behavior of GNR can be briefly explained by the contradictory effect of the vibrational force on the VDW force. A local VDW attractive force of the carbon clusters that is smaller than the sum of their vibrational and elastic forces results in a shorter distance between GNR sheets leading to a decrease of the sheet resistance of the GNR film. The opposite case results in an increase of the sheet resistance of the film due to the shortening of the local distance of carbon clusters.

To illustrate the VDW interaction between GNR sheets, we prepared a vacuum XRD holder with a ceramic heater, and obtained vacuum XRD spectra of the samples at various temperatures. This was done because we can assume that the distance between GNR sheets should be changed by VDW interaction when vacuum pressure is applied. The temperature of the holder was adjusted to 40 °C, 70 °C, and 100 °C under vacuum pressure (approximately 0.3 Torr), and the XRD spectra of the sample were obtained and compared with the results measured at room temperature under atmospheric pressure and vacuum pressure (approximately 0.3 Torr). The XRD spectra in Fig. 5a–c clearly demonstrate that the major XRD peaks of the sample (approximately 17°, 17°, and 19° for RGO, GNR0, and GNR3, respectively) shifted. The peak intensity also decreased because of the Debye-Waller temperature factor associated with atomic vibrations.^18^ Notably, a bi-directional shift of the main peak can be observed in the XRD spectra measured at room temperature under vacuum -the right-shift for RGO (an approximately 0.75 ° shift corresponding to a 0.022 nm shift; see Supplementary Table S1) and left-shift for GNR3 (an approximately 1.5 ° shift corresponding to a 0.041 nm shift). The opposite shift directions of the main peak indicate that the compressive force of the RGO sample is dominant over the tensile force, whereas in GNR3, the tensile force is greater because of the one-dimensional structure of it. With increasing temperature, the main XRD peaks of RGO and GNR0 moved to higher 2θ angles, whereas the peak position of GNR0 did not change. Based on the hypothesis for Fig. 1b, the change of the local distance between carbon clusters on adjacent RGO sheets should create nonuniform strain in the RGO sheet leading to broadening of the main peaks due to the combination of the two forces in the sample.^19^ This result matches well with our hypothesis that provides the tensile and compressive forces at high temperature in Fig. 1b. At room temperature, the one-direction shift of the main peak also supports our hypothesis for Fig. 1a. Based on the XRD results, the change of distance of each sample with vacuum pressure was compared with theoretical simulation of the electrical behavior of RGO with respect to pressure. According to theoretical calculations,^9^ the distance between RGO sheets changes from 0.023 nm at 50 °C to 0.026 nm at 100 °C for a pressure change of 0.3 to 1 Torr due to VDW force (See Figure S3). This value is larger than the actual distance change obtained from XRD spectra in Fig. 4, but very similar.

To evaluate the response time and repeatability of the pressure reading at 30 °C, the chamber was depressurized to approximately 5 × 10^−3^ Torr and then leaked it completely. During repetitions of this process, the sheet resistance of the sample was simultaneously recorded. The results demonstrate the high durability of the GNR pressure sensor because it could withstand pressure shocks from high to low pressure or from instantaneous pressure changes from low to high pressure (See Fig. 5d–f). The response of the GNR sensor to pressure changes was rapid (in seconds). However, the resistance of the sample could take several minutes to reach a steady state. There are two main factors contributing to the tail end of the response time: the diffusion rate of gas molecules in the sample as discussed in the Introduction, and the interaction between the gas molecules in the sample and the oxygen residue. In this study, although we could not estimate the effect of the oxygenated sites of the sample on the response time, the normalized response time in Fig. 5g indicates that the shortened diffusion path of gas molecules can decrease the tailing of the response time. This result implies that the response time of graphene sensors can be improved by the modification of the graphene structure. Figure 5h shows the sensitivities of the samples at 30 °C between 0.001 and ambient pressure (approximately 760 Torr). The sensitivity of the sample was compared using the rate of resistance change (RRC) in the given pressure range: RRC (%) = 100 × (R_max_ − R_min_)/R_max_, where R_max_ and R_min_ are the maximum and minimum sheet resistances in the pressure range. In Fig. 5h, GNR3 exhibited the highest RRC against pressure change, and its RRC value was three times higher RRC than that of RGO in the given pressure range. In Table S2, the sensitivity of GNR3 is compared with those of previously reported pressure sensors using carbonaceous materials. Except for the pressure sensor using resonance frequencies, the sensitivity of GNR3 was much larger than those of popular pressure sensors using piezoresistivity.^2–5^ We also measured the sensor activity of GNR3 in the pressure range between 10^−3^ to 8 × 10^−7^ Torr. Although the detailed results are not provided in this report because of the ions produced by the hot cathode ion-gauge interrupted the pressure reading of the GNR3 sensor, Fig. 5i shows that the GNR3 sensor worked well up to the highest chamber vacuum of 8 × 10^−7^ Torr.

In summary, GNR mixtures synthesized using methods previously reported in the literature contained GNRs of various chemical structures and sizes. Using this mixture, we showed that several different GNR samples could be separated by controlling the pH of the mixture, even though they had almost the same oxygen contents. The sheet resistances of sensor devices prepared using the GNR samples decreased with decreasing pressure until reaching a pressure between approximately 1 and 10 Torr. Further reduction of the pressure resulted in different electrical behavior depending on temperature. The sheet resistance of the GNR film gradually decreased with decreasing pressure at 30 °C, whereas it increased at 100 °C. To explain this result, we introduced the hypothesis of a local or wide effect of VDW interactions on carbon clusters of GNR sheets. A smaller local VDW attractive force of the carbon clusters than the sum of their vibrational and elastic forces resulted in a shorter distance between GNR sheets, leading to a decrease of sheet resistance of the GNR film. The opposite case resulted in an increase of the sheet resistance of the GNR sample due to shortening of the local distance of carbon clusters. To prove this hypothesis, we measured vacuum XRD and observed that the major XRD peaks of the sample shifted approximately 0.75° for RGO (corresponding to a 0.022 nm shift) and approximately 1.5° for GNR3 (corresponding to a 0.041 nm shift). The shift distance for RGO was similar to that obtained theoretically. In addition, the shift direction of the main peak indicated that the compressive force of the RGO sample was dominant and that for GNR3, the tensile force was dominant under vacuum pressure. The response of the GNR sensor to pressure changes was rapid (in seconds). The normalized response time of each sample proved that the sensor using GNR3 decreased the tailed response time because of the shortened diffusion path of gas molecules. The sensitivity of GNR3 was three times greater than that of RGO in the given pressure range. Moreover, the sensitivity of the GNR3 sensor was much larger than those of most popular pressure sensors using Piezoresistivity (except for the pressure sensor using resonance frequencies). The GNR3 sensor was able to read vacuum pressures up to 8 × 10^−7^ Torr in this experiment and is expected to be able to read high vacuum pressures with a high durability to pressure shocks.

## Methods

### Preparation of GO

We obtained GO from synthetic graphite (<20 μm; Aldrich) using the modified Hummers method.^14^

### Preparation of GNR

The GNR was fabricated by modifying a previously reported synthesis method.^15^ First, 1 g of multiwalled CNTs (MWCNTs) (PlasmaChem GmbH, number of ring: 3 ~ 15, length: 1 ~ 10 μm, purity: >95%) were dispersed in a mixture of 150 ml of H_2_SO_4_ (98%) and 20 ml of H_3_PO_4_ (85%) in an ice bath. Then, 12 g of KMnO_4_ (Aldrich) was slowly added to the reaction mixture. After 10 min, the mixture was heated to 45 °C and stirred for 24 h. The reaction was terminated by slowly adding 300 ml of distilled water for 30 min, and then, H_2_O_2_ (Dae Jung, 30 wt%) was added until the bubbles in the mixtures disappeared. The oxidized CNTs were centrifuged and cleaned three times with 5% HCl. Then, the residues were dispersed in 100 ml of 3% HCl before being centrifuged. The supernatant containing the GNR was transferred and stored in vials. We performed these processes repeatedly until the supernatant obtained a bright yellowish brown color. Similar processes were performed for 2%, 1%, and 0% HCl. The samples were designated as GNR3 for 3% HCl, GNR2 for 2% HCl, GNR1 for 1% HCl, and GNR0 for 0% HCl. Each collected sample containing GNR was transferred to a dialysis tube and dialyzed 40 times with distilled water for two weeks to remove any impurities.

### Characterizations

The samples were analyzed using X-ray photoelectron spectroscopy (XPS; VG Scientific) transmission electron microscopy (TEM; JEOL, JEM-2200FS with image Cs-compensator), Fourier transform infrared (FT-IR) spectroscopy (Shimadzu, IRTracer-100), Raman spectroscopy (Horiba, LabRam HR), and XRD (Rigaku, D/MAX 2500). The XRD patterns were obtained under various conditions using a vacuum and heatable sample holder.

### Fabrication of a test device and measurement of pressure sensor activity

First, 5 ml of a 0.01 wt% GNR sample was mixed with 1 ml of 8 wt% hydrazine solution. Polyethlene terephthalate (PET) tape (50 μm thick, waterproof) with a 3-mm-diameter hole was attached to the center of the indium tin oxide electrode (ITO) with a gap of approximately 0.3 μm, and 0.003 ml of the mixture was dropped into the hole. The detailed structure of the sensor device is shown in Figure S4. Thereafter, the film was dried at 60 °C, the PET tape was removed, and then the film was heat-treated at 200 °C for 1 h in air, and at 450 °C for 1 h under vacuum. To investigate the sensor activity, the sample was placed on a heating plate in a vacuum chamber with a four-point probe. The sensor activities of the GNR0, GNR3 and RGO (reference) samples were investigated for comparison. Before measurement, the samples were first heat-treated for 30 min at 100 °C, and then, the sheet resistance was measured at 30, 50, 75, and 100 °C. The probe measured the sheet resistances at 1.67 s intervals against elevated pressure in vacuum under a constant leakage of approximately 3 × 10^−3^ Torr/min until the pressure reached 1 Torr. Above 1 Torr, we manually increased the pressure and then measured the sheet resistances of the samples. The vacuum pressure was measured by a calibrated capacitor gauge with a maximum error of 0.3%.

## Electronic supplementary material

Supplementary information
