# Supplementary material for: Ultra-sensitive graphene sensor for measuring high vacuum pressure
Source: Sci Rep. 2017 Oct 3;7:12604. doi: 10.1038/s41598-017-13038-3 (PMC5626739; doi:10.1038/s41598-017-13038-3)
Supplement: Supplementary file 1 — Supplementary information [file 41598_2017_13038_MOESM1_ESM.doc]

**Supplementary Information**

Ultra-sensitive graphene sensor for measuring high vacuum pressure

Sung Il Ahn^*^, Ju Ra Jung, So Young Choi, Min Hwa Son, Yu Jin Hong, and Jung-Chul Park

Division of Energy and Chemical Engineering, Silla University, Busan 617-736 (Republic of Korea)

**1. Commercial vacuum gauges**

**2. TEM images of concentrated GNR samples**

**3. Theoretical calculation of sensor behavior of RGO**

**4. Sensor device structure**

**5. Calculated d (nm) and Δd (nm) values at the peak maximum of the XRD spectra**

**6. Sensitivity comparison**

.

**1. Commercial vacuum gauges**

Figure S1. Pressure reading range of selected commercial vacuum gauge. The pressure range is referenced to the pressure gauge of several vacuum gauge companies.

**2. TEM images of concentrated GNR samples**

Figure S2. TEM images of a. GNR0, b. GNR1, c. GNR2, and d. GNR3 from concentrated GNR samples

**3. Theoretical calculation of sensor behavior of RGO**

Figure S3 Theoretically calculated shift distance of RGO

Figure S3 presents P vs
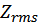
 (RMS value of the average position of the axial layer) graphs for various temperatures based on previous experimental data.^9^ Based on the graphs, we calculated
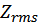
 at 0.3 Torr and compared this value with the shift distance of the main XRD peak of RGO under vacuum (0.3 Torr) in the manuscript.

**4. Sensor device structure**

Figure S4. Structure of sensor device used in this study.

**5.** **Calculated d (nm) and Δd (nm) values at the peak maximum of the XRD spectra**

Table S1. Calculated d (nm) and Δd (nm) values at the maximum of the X-ray diffraction (XRD) patterns in Figure 4a-c (HWHM_L_: Half width at half maximum at a lower angle, HWHM_H_: Half width at half maximum at a higher angle).

**6. Sensitivity comparison**

Table S2. Comparison of GNR3 sensitivity with those of previously reported pressure sensors.
